# Susceptibility to Transmitting HIV in Patients Initiating Antiretroviral Therapy in Rural District Hospitals in Cameroon (Stratall ANRS 12110/ESTHER Trial)

**DOI:** 10.1371/journal.pone.0062611

**Published:** 2013-04-30

**Authors:** Gilbert Ndziessi, Julien Cohen, Charles Kouanfack, Fabienne Marcellin, Maria Patrizia Carierri, Gabrièle Laborde-Balen, Camélia Protopopescu, Avelin Fobang Aghokeng, Jean-Paul Moatti, Bruno Spire, Eric Delaporte, Christian Laurent, Sylvie Boyer, for the Stratall ANRS 12110 / ESTHER Study Group, M. Biwolé-Sida, C. Kouanfack, S. Koulla-Shiro, A. Bourgeois, E. Delaporte, C. Laurent, M. Peeters, G. Laborde-Balen, M. Dontsop, S. Kazé, J-M. Mben, A. Aghokeng, M.G. Edoul, E. Mpoudi-Ngolé, M. Tongo, S. Boyer, M.P. Carrieri, F. Marcellin, J-P. Moatti, B. Spire, C. Abé, S-C. Abega, C-R. Bonono, H. Mimcheu, S. Ngo Yebga, C. Paul Bile, S. Abada, T. Abanda, J. Baga, P. Bilobi Fouda, P. Etong Mve, G. Fetse Tama, H. Kemo, A. Ongodo, V. Tadewa, HD. Voundi, A. Ambani, M. Atangana, J-C. Biaback, M. Kennedy, H. Kibedou, F. Kounga, M. Maguip Abanda, E. Mamang, A. Mikone, S. Tang, E. Tchuangue, S. Tchuenko, D. Yakan, J. Assandje, S. Ebana, D. Ebo’o, D. Etoundi, G. Ngama, P. Mbarga Ango, J. Mbezele, G. Mbong, C. Moung, N. Ekotto, G. Nguemba Balla, G. Ottou, M. Tigougmo, R. Beyala, B. Ebene, C. Effemba, F. Eyebe, M-M. Hadjaratou, T. Mbarga, M. Metou, M. Ndam, B. Ngoa, EB. Ngock, N. Obam, A. M. Abomo, G. Angoula, E. Ekassi, Essama, J.J. Lentchou, I. Mvilongo, J. Ngapou, F. Ntokombo, V. Ondoua, R. Palawo, S. Sebe, E. Sinou, D. Wankam, I. Zobo, B. Akono, A. L. Ambani, L. Bilock, R. Bilo’o, J. Boombhi, F.X. Fouda, M. Guitonga, R. Mad’aa, D.R. Metou’ou, S. Mgbih, A. Noah, M. Tadena, Ntcham, G. Ambassa Elime, A.A. Bonongnaba, E. Foaleng, R.M. Heles, R. Messina, O. Nana Ndankou, S.A. Ngono, D. Ngono Menounga, S.S. Sil, L. Tchouamou, B. Zambou, R. Abomo, J. Ambomo, C. Beyomo, P. Eloundou, C. Ewole, J. Fokom, M. Mvoto, M. Ngadena, R. Nyolo, C. Onana, A. Oyie, P. Antyimi, S. Bella Mbatonga, M. Bikomo, Y. Molo Bodo, S. Ndi Ntang, P. Ndoudoumou, L. Ndzomo, S.O. Ngolo, M. Nkengue, Nkoa, Y. Tchinda

**Affiliations:** Central hospital, Yaoundé, Cameroon; Central hospital, Yaoundé, Cameroon; Central hospital, Yaoundé, Cameroon; IRD, University Montpellier 1, UMI 233, Montpellier, France; IRD, University Montpellier 1, UMI 233, Montpellier, France; IRD, University Montpellier 1, UMI 233, Montpellier, France; IRD, University Montpellier 1, UMI 233, Montpellier, France; French Ministry of Foreign Affairs, Yaoundé, Cameroon; IRD, Yaoundé, Cameroon; IRD, Yaoundé, Cameroon; IRD, Yaoundé, Cameroon; Virology Laboratory, IMPM/CREMER/IRD-UMI 233, Yaoundé, Cameroon; Virology Laboratory, IMPM/CREMER/IRD-UMI 233, Yaoundé, Cameroon; Virology Laboratory, IMPM/CREMER/IRD-UMI 233, Yaoundé, Cameroon; Virology Laboratory, IMPM/CREMER/IRD-UMI 233, Yaoundé, Cameroon; INSERM, IRD, University Marseille, UMR 912, Marseille, France; INSERM, IRD, University Marseille, UMR 912, Marseille, France; INSERM, IRD, University Marseille, UMR 912, Marseille, France; INSERM, IRD, University Marseille, UMR 912, Marseille, France; INSERM, IRD, University Marseille, UMR 912, Marseille, France; IRSA, Catholic University of Central Africa, Yaoundé, Cameroon; IRSA, Catholic University of Central Africa, Yaoundé, Cameroon; IRSA, Catholic University of Central Africa, Yaoundé, Cameroon; IRSA, Catholic University of Central Africa, Yaoundé, Cameroon; IRSA, Catholic University of Central Africa, Yaoundé, Cameroon; IRSA, Catholic University of Central Africa, Yaoundé, Cameroon; District Hospital, Ayos, Cameroon; District Hospital, Ayos, Cameroon; District Hospital, Ayos, Cameroon; District Hospital, Ayos, Cameroon; District Hospital, Ayos, Cameroon; District Hospital, Ayos, Cameroon; District Hospital, Ayos, Cameroon; District Hospital, Ayos, Cameroon; District Hospital, Ayos, Cameroon; District Hospital, Ayos, Cameroon; District Hospital, Bafia, Cameroon; District Hospital, Bafia, Cameroon; District Hospital, Bafia, Cameroon; District Hospital, Bafia, Cameroon; District Hospital, Bafia, Cameroon; District Hospital, Bafia, Cameroon; District Hospital, Bafia, Cameroon; District Hospital, Bafia, Cameroon; District Hospital, Bafia, Cameroon; District Hospital, Bafia, Cameroon; District Hospital, Bafia, Cameroon; District Hospital, Bafia, Cameroon; District Hospital, Bafia, Cameroon; District Hospital, Mbalmayo, Cameroon; District Hospital, Mbalmayo, Cameroon; District Hospital, Mbalmayo, Cameroon; District Hospital, Mbalmayo, Cameroon; District Hospital, Mbalmayo, Cameroon; District Hospital, Mbalmayo, Cameroon; District Hospital, Mbalmayo, Cameroon; District Hospital, Mbalmayo, Cameroon; District Hospital, Mbalmayo, Cameroon; District Hospital, Mbalmayo, Cameroon; District Hospital, Mbalmayo, Cameroon; District Hospital, Mbalmayo, Cameroon; District Hospital, Mbalmayo, Cameroon; District hospital, Mfou, Cameroon; District hospital, Mfou, Cameroon; District hospital, Mfou, Cameroon; District hospital, Mfou, Cameroon; District hospital, Mfou, Cameroon; District hospital, Mfou, Cameroon; District hospital, Mfou, Cameroon; District hospital, Mfou, Cameroon; District hospital, Mfou, Cameroon; District hospital, Mfou, Cameroon; District hospital, Mfou, Cameroon; District hospital, Monatélé, Cameroon; District hospital, Monatélé, Cameroon; District hospital, Monatélé, Cameroon; District hospital, Monatélé, Cameroon; District hospital, Monatélé, Cameroon; District hospital, Monatélé, Cameroon; District hospital, Monatélé, Cameroon; District hospital, Monatélé, Cameroon; District hospital, Monatélé, Cameroon; District hospital, Monatélé, Cameroon; District hospital, Monatélé, Cameroon; District hospital, Monatélé, Cameroon; District hospital, Monatélé, Cameroon; District hospital, Monatélé, Cameroon; District hospital, Nanga Eboko, Cameroon; District hospital, Nanga Eboko, Cameroon; District hospital, Nanga Eboko, Cameroon; District hospital, Nanga Eboko, Cameroon; District hospital, Nanga Eboko, Cameroon; District hospital, Nanga Eboko, Cameroon; District hospital, Nanga Eboko, Cameroon; District hospital, Nanga Eboko, Cameroon; District hospital, Nanga Eboko, Cameroon; District hospital, Nanga Eboko, Cameroon; District hospital, Nanga Eboko, Cameroon; District hospital, Nanga Eboko, Cameroon; District hospital, Nanga Eboko, Cameroon; District hospital, Ndikinimeki, Cameroon; District hospital, Ndikinimeki, Cameroon; District hospital, Ndikinimeki, Cameroon; District hospital, Ndikinimeki, Cameroon; District hospital, Ndikinimeki, Cameroon; District hospital, Ndikinimeki, Cameroon; District hospital, Ndikinimeki, Cameroon; District hospital, Ndikinimeki, Cameroon; District hospital, Ndikinimeki, Cameroon; District hospital, Ndikinimeki, Cameroon; District hospital, Ndikinimeki, Cameroon; District hospital, Obala, Cameroon; District hospital, Obala, Cameroon; District hospital, Obala, Cameroon; District hospital, Obala, Cameroon; District hospital, Obala, Cameroon; District hospital, Obala, Cameroon; District hospital, Obala, Cameroon; District hospital, Obala, Cameroon; District hospital, Obala, Cameroon; District hospital, Obala, Cameroon; District hospital, Obala, Cameroon; District hospital, Sa’a, Cameroon; District hospital, Sa’a, Cameroon; District hospital, Sa’a, Cameroon; District hospital, Sa’a, Cameroon; District hospital, Sa’a, Cameroon; District hospital, Sa’a, Cameroon; District hospital, Sa’a, Cameroon; District hospital, Sa’a, Cameroon; District hospital, Sa’a, Cameroon; District hospital, Sa’a, Cameroon; District hospital, Sa’a, Cameroon; 1 INSERM, UMR912 (SESSTIM), Marseille, France; 2 Aix Marseille Université, UMR_S912, IRD, Marseille, France; 3 ORS PACA, Observatoire Régional de la Santé Provence-Alpes-Côte d'Azur, Marseille, France; 4 Central Hospital, UMI 233, Yaoundé, Cameroon; 5 UMI 233, French Ministry of Foreign Affairs, Yaoundé, Cameroon; 6 Institut de Recherche pour le Développement (IRD), University Montpellier 1, UMI 233, Montpellier, France; 7 Virology laboratory IRD/IMPM/CREMER, Yaoundé, Cameroon; 8 Department of Infectious and Tropical Diseases, University Hospital, Montpellier, France; British Columbia Centre for Excellence in HIV/AIDS, Canada

## Abstract

**Objectives:**

Using cohort data nested in a randomized trial conducted in Cameroon, this study aimed to investigate time trends and predictors of the susceptibility to transmitting HIV during the first 24 months of treatment.

**Methods:**

The outcome, susceptibility to transmitting HIV, was defined as reporting inconsistent condom use and experiencing incomplete virological suppression. Mixed logistic regressions were performed to identify predictors of this outcome among 250 patients reporting to have had sexual relationships either with HIV-negative or unknown HIV status partner(s).

**Results:**

Despite an initial decrease from 76% at M0 to 50% at M6, the rate of inconsistent condom use significantly increased from M12 (59%) to M24 (66%) (p = 0.017). However, the proportion of patients susceptible to transmitting HIV significantly decreased over follow-up from 76% at M0, to 50% at M6, 31% at M12 and 27% at M24 (p<0.001). After controlling for age, gender and intervention group, we found that perceiving healthcare staff’s readiness to listen as poor (adjusted odds ratios (AOR) [95% Confidence Interval (CI)] = 1.87 [1.01–3.46]), reporting to have sexual relationships more than once per week (AOR [95%CI] = 2.52 [1.29–4.93]), having more than one sexual partner (AOR [95%CI] = 2.53 [1.21–5.30]) and desiring a/another child (AOR [95%CI] = 2.07 [1.10–3.87]) were all associated with a higher risk of being susceptible to transmitting HIV. Conversely, time since ART initiation (AOR [95%CI] = 0.66 [0.53–0.83] for an extra 6 months and ART adherence (AOR [95%CI] = 0.33 [0.15–0.72]) were significantly associated with a lower risk of being susceptible to transmitting HIV.

**Conclusions:**

The decrease observed in the susceptibility to transmitting HIV suggests that fear of behavioural disinhibition should not be a barrier to universal access to ART. However, developing adequate preventive interventions matching patients’ expectations -like the desire to have children- and strengthening healthcare staff’s counselling skills are urgently needed to maximize the impact of ART in slowing the HIV epidemic.

## Introduction

The number of new HIV infections remains high worldwide and especially in sub-Saharan Africa where 1.8 million new infections occurred in 2011, accounting for 70% of the total number of new infections in the world [1]. While it is now acknowledged that a large range of contextual, behavioural and virological factors plays a role in the dynamic of the HIV epidemic, sexual relationships remain the main driver of HIV transmission in the region [2].

Over the past ten years, antiretroviral therapy (ART) has become available for an ever-growing number of people living with HIV/AIDS (PLWHA) with 56% of those in need of treatment having access to it in 2011 as opposed to only 3% in 2003 [1], [3]. Besides its individual clinical benefits in terms of mortality and morbidity reduction, recent evidence suggests that treatment can dramatically reduce sexual transmission of HIV by suppressing viral levels in the HIV-infected partner’s genital tract. The “Swiss statement” first established in 2008 that the risk of sexual HIV transmission is negligibly low in ART-treated patients with high adherence level, undetectable viral load for more than 6 months and no concurrent sexually transmitted infections [4]. Subsequently, several other studies consistently shown that, thanks to ART, HIV sexual transmission in serodiscordant partners not using condoms is dramatically reduced when the viral load of the infected partner is maintained at low or undetectable levels [5]. Lastly, recent evidence from the HIV Prevention Trials Network (HPTN052) suggests that early HIV diagnosis followed by immediate ART regardless of immunological status can reduce the risk of HIV sexual transmission in serodiscordant couples by 96% [6].

Based on this evidence, ART is increasingly considered as a powerful prevention tool to slow HIV epidemics in countries where sexual transmission is predominant. The impact of ART on the future course of the HIV epidemic will however depend on several factors among which controlling viremia and reducing risky sexual behaviours are key. A number of cross-sectional studies and observational cohorts have been conducted in sub-Saharan Africa to assess risky sexual behaviours among PLWHA initiating ART [7]–[20]. They showed consistent decreases in unprotected sex after ART initiation [21], [22] but did not simultaneously investigate patients’ viremia levels and condom use. However, estimating the proportion of viremic PLWHA with risky sexual behaviours and identifying the characteristics of those individuals is crucial for maximizing the impact of ART on the reduction of HIV incidence in the sub-Saharan African setting.

Using cohort data nested in the Stratall ANRS 12110/ESTHER trial in which Cameroonian patients initiating ART were followed-up for 24 months, this study aimed i) to describe time trends of sexual risk behaviours and HIV transmission risk over the first 24 months of treatment; ii) to identify predictors of the susceptibility to transmitting HIV defined as reporting inconsistent condom use and experiencing incomplete virological suppression.

## Materials and Methods

### Study Design and Procedures

Longitudinal data collection, including psychosocial and sexual behavioural data, was nested with*in* the randomized, 24-month open-label Stratall trial designed to compare the effectiveness of clinical monitoring alone (CLIN group, n = 238 patients) versus clinical and laboratory monitoring of viral load and CD4 cell count (LAB group, n = 221 patients). The detailed methodology and main results of the trial have been reported elsewhere [23]. Briefly, 459 ART-naive patients were enrolled between May 23, 2006 and January 31, 2008 in nine rural district hospitals located in the Centre Region of Cameroon and followed-up over 24 months. Eligibility criteria included being infected with HIV-1, being older than 18 years and being eligible for ART, in accordance with national guidelines and WHO 2006 recommendations [24]. Participants were requested to attend clinical visits, performed by healthcare workers involved in routine activities (either doctors or nurses), at weeks 0 and 2, months 1 and 3, and every three months thereafter, until the 24th month. CD4 cell count (FACSCount device, Becton Dickinson, Mountain View, CA, USA) and plasma viral load (RealTime HIV-1 assay, Abbott Molecular, Des Plaines, IL, USA) were measured at baseline and every six months thereafter. All results for participants in the LAB group were returned immediately to doctors. Results for participants in the CLIN group were retained at the reference laboratory until month 24. Patients also received counselling on adherence to ART and ways to prevent HIV transmission at entry into care and, if requested, also during follow-up. Condoms were provided free of charge in the hospitals’ pharmacies.

Socio-economic, psychosocial and behavioural data were collected after the clinical visit at baseline (M0, i.e. the date of treatment initiation), and at months 6, 12 and 24. Anonymous face-to-face questionnaires were administered by community health workers in a private room thereby ensuring confidentiality. The latter were not part of the medical team and their primary role was to improve linkage to care. They were trained by the research team to use non-judgmental approaches during interviews so that patients would feel as comfortable as possible when answering questions. A series of questions was asked to collect information about adherence to ART, patient-caregiver relationships, sexual behaviours and perceived health.

The study protocol was approved by the National Ethics Committee of Cameroon and the institutional Ethics Committee of the Institut de Recherche pour le Développement (France). All study participants provided written informed consent.

### Variables

Variables retained for the analyses were classified into the following categories:

- socio-demographic and economic characteristics: age, gender, educational level (primary school or lower versus higher than primary school); living in a couple (updated at each administered questionnaire); perceived social level (assessed using a ten-point visual scale, with higher scores denoting a better perceived social situation (higher income, higher educational level and better job)) [25].
- Patient-caregiver relationships: trust in physicians, trust in other staff involved in clinical visits (both assessed using a three-point Likert scale (no trust, little trust and complete trust)); perception of the healthcare staff’s readiness to listen (assessed using a six-point visual scale ranging from 1 to 6 with higher values denoting a high perceived degree of readiness to listen [26].
- Reproductive life and sexual behaviours during the previous three months: having biological children, desire to have a/another child; number of sexual partners including casual partners (1, 2–4, 5–10, >10), frequency of sexual relationships (less than once a month, once a month, several times a month, several times a week, daily); knowledge of the HIV status of one’s main sexual partner (HIV-negative, HIV-positive, unknown); condom use assessed separately for the main partner and for casual partners using a four-point Likert scale (never, sometimes, nearly always, always) and history of forced sexual relationships.
- Psychosocial variables: binge drinking defined as drinking on one occasion at least three large bottles of beer (i.e. 2 litres with 5.2 ml of alcohol per 100 ml) and/or six glasses or more of other alcoholic beverages [27]; experiencing depressive symptoms, assessed using the Center for Epidemiological Studies Depression Scale (CES-D) and defined as obtaining a CES-D score >16 (on a scale from 0 to 60) [28].
- Biological, clinical and ART-related characteristics: WHO clinical stage, CD4 cell count, viral load, intervention group (clinical versus clinical and laboratory monitoring), time since ART initiation, reported number of side effects during the previous 4 weeks (assessed by the HIV symptom index developed by Justice which includes the 20 most frequent ART-related symptoms such as nausea, sexual disorders, etc. [29]) and adherence to ART, assessed using a validated scale [30], [31].

### Definition of Inconsistent Condom Use, Incomplete Virological Suppression and Susceptibility to Transmitting HIV

Inconsistent condom use was defined as reporting to have “never”, “sometimes” or “nearly always” used condoms with one’s partner(s) - either HIV-negative or of unknown HIV status - during the previous three months [14], [15], [32], [33]. As information about HIV-status was only available for the main partner, all casual partners were considered to have unknown HIV status.

Incomplete virological suppression at a given visit was defined as having had at least one detectable viral load (≥40 copies/ml) during the previous six months. This variable was only defined from M6 onwards as patients were not yet treated at enrolment (i.e. M0) and all had a detectable viral load.

Susceptibility to transmitting HIV at a given visit was defined both by self-reported inconsistent condom use and by incomplete virological suppression, except at M0, where all patients were considered susceptible to transmitting HIV if they reported inconsistent condom use.

Both the variables (i.e. inconsistent condom use and viral load used to define incomplete virological suppression) were measured simultaneously at the same time points.

### Statistical Analysis

The study population included patients reporting at least once during follow-up that they had sexual relationships either with HIV-negative or unknown HIV-status partner(s). Both CLIN and LAB groups were pooled in the analyses as the proportion of patients susceptible to transmitting HIV was not significantly different over the whole follow-up period between the two groups (p = 0.45). We first described time trends of inconsistent condom use, incomplete virological suppression and susceptibility to transmitting HIV over the first 24 months of ART. McNemar tests were used to assess whether the time trends observed were significant. Mixed-effect logistic regressions – which enable the correlation between repeated measures to be taken into account [34] - were used to identify the predictors of the susceptibility to transmitting HIV. Variables with a *p-*value lower than 0.20 in univariate analyses were considered eligible to enter the initial multivariate model (i.e. living in a couple, perceiving one’s own social level as low, perceiving healthcare staff’s readiness to listen as poor, reporting to have sexual relationships more than once per week, having more than one sexual partner, desiring to have a/another child, experiencing depressive symptoms, total number of ART-related symptoms, time since ART initiation and adherence to ART).

The final multivariate model was obtained using a backward stepwise selection procedure based on the log-likelihood ratio test to eliminate non-significant variables (p>0.05) from the initial model. In addition, the intervention group variable was kept in the final model to control for differences in care and clinical follow-up as well as gender and age to control for key socio-demographic characteristics.

Statistical analyses were performed using SPSS v15.0 (SPSS, Inc., Chicago, Illinois, USA) and Intercooled Stata 10 (StataCorp LP, College Station, Texas, USA) software packages.

## Results

### Study Population

Over the whole follow-up, 291 (63%) patients among the 459 enrolled in the Stratall trial reported at least once that they had sexual relationships (irrespective of the partner(s)’ HIV status) and 261 (57%) reported at least once that they had sexual relationships with a HIV-negative partner(s) or partner(s) with unknown HIV status (i.e. a total of 505 visits). Among the latter, 250 patients, accounting for a total of 473 visits (i.e. 114 at M0, 112 at M6, 115 at M12 and 132 at M24), had complete data on condom use and viral load (study population).

### Main Characteristics of the Study Population at Enrolment and Over Follow-up

At enrolment, median [interquartile range, IQR] age was 35 [29]–[42] years, 179 (72%) patients were women, 85 (34%) were living in a couple, 202 (81%) had children and 27 (11%) reported the desire to have a/another child. The socioeconomic status of the study population was low with 129 (52%) patients having an educational level higher than primary school and 89 (36%) perceiving their social level as low. In addition, 161 (64%) patients reported depressive symptoms, 36 (14%) binge drinking and 66 (26%) were at WHO clinical stage 4.

CD4 cell count significantly increased over follow-up from a median [IQR] of 183 [81–361] cells/mm^3^ at enrolment to 369 [224–552] cells/mm^3^ at M6, 370 [245–581] cells/mm^3^ at M12 and 428 [284–615] cells/mm^3^ at M24 (p<0.001). Furthermore, over the whole follow-up, 153 (61.2%) patients reported having sex only with their main partner, 96 (38.4%) with their main and with a casual partner(s) and 1 (0.4%) only with a casual partner(s).

### Time Trends of Inconsistent Condom Use, Incomplete Virological Suppression and Susceptibility to Transmitting HIV

**Figure 1:**
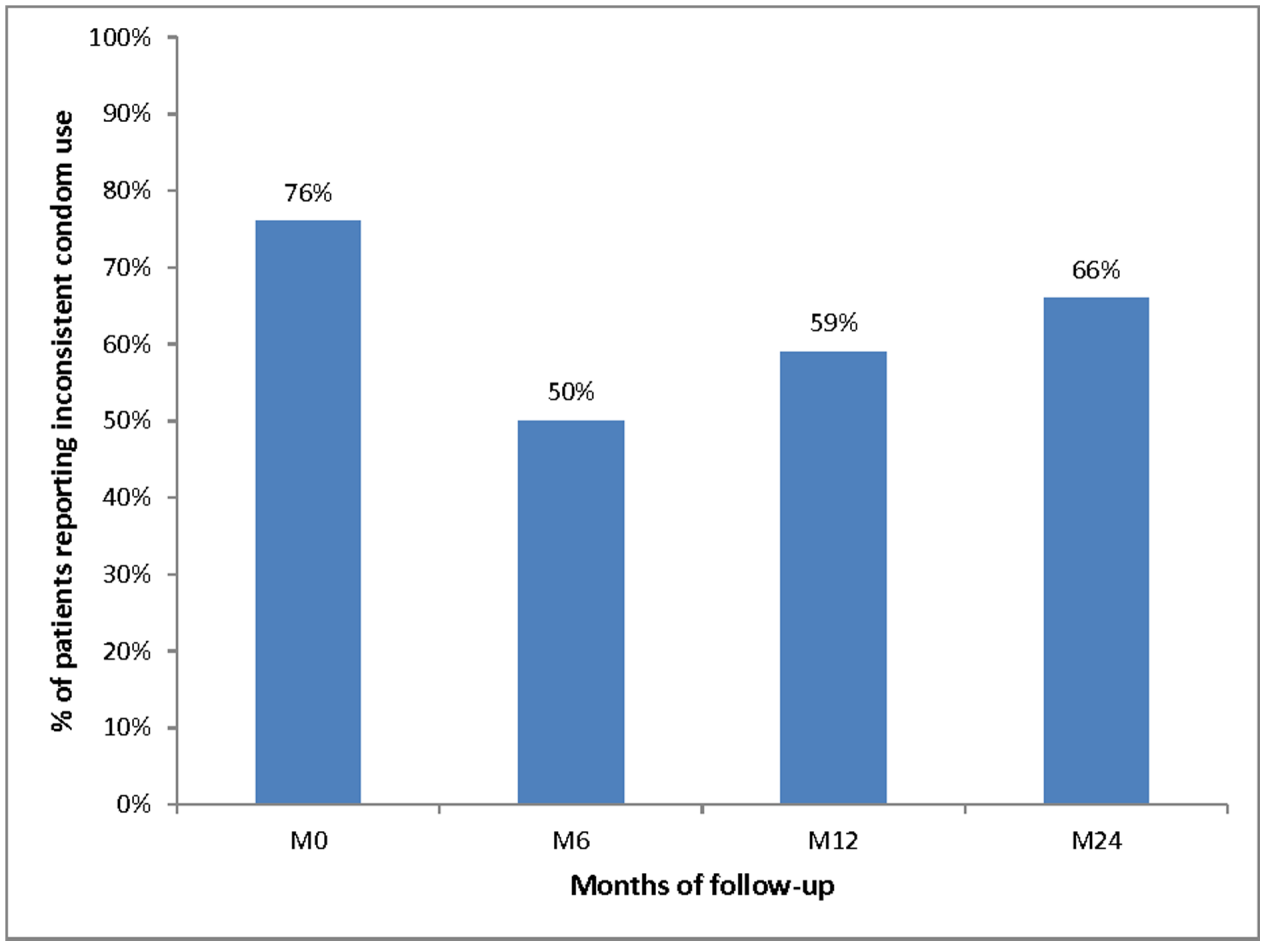
Time trend of the proportion of patients reporting inconsistent condom use with sexual partners either HIV-negative or of unknown HIV status over the first 24 months of ART (Stratall ANRS 12110/ESTHER trial, 250 patients, 473 visits).

**Figure 2:**
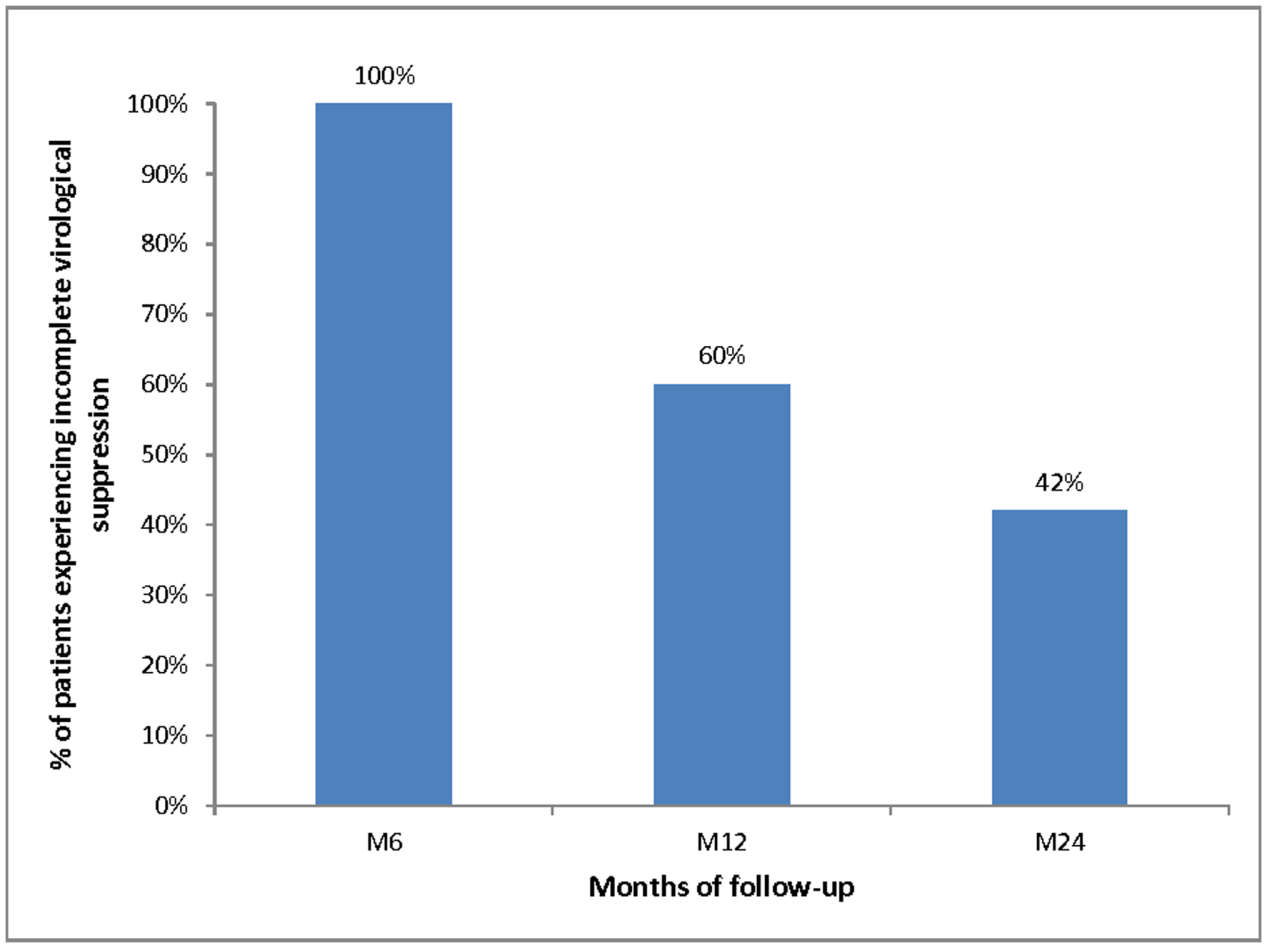
Time trend of the proportion of patients with incomplete virological suppression (defined as having had a least one detectable viral load (≥40 copies/ml) during the previous 6 months) over the first 24 months (Stratall ANRS 12110/ESTHER trial, 250 patients, 473 visits).

**Figure 3:**
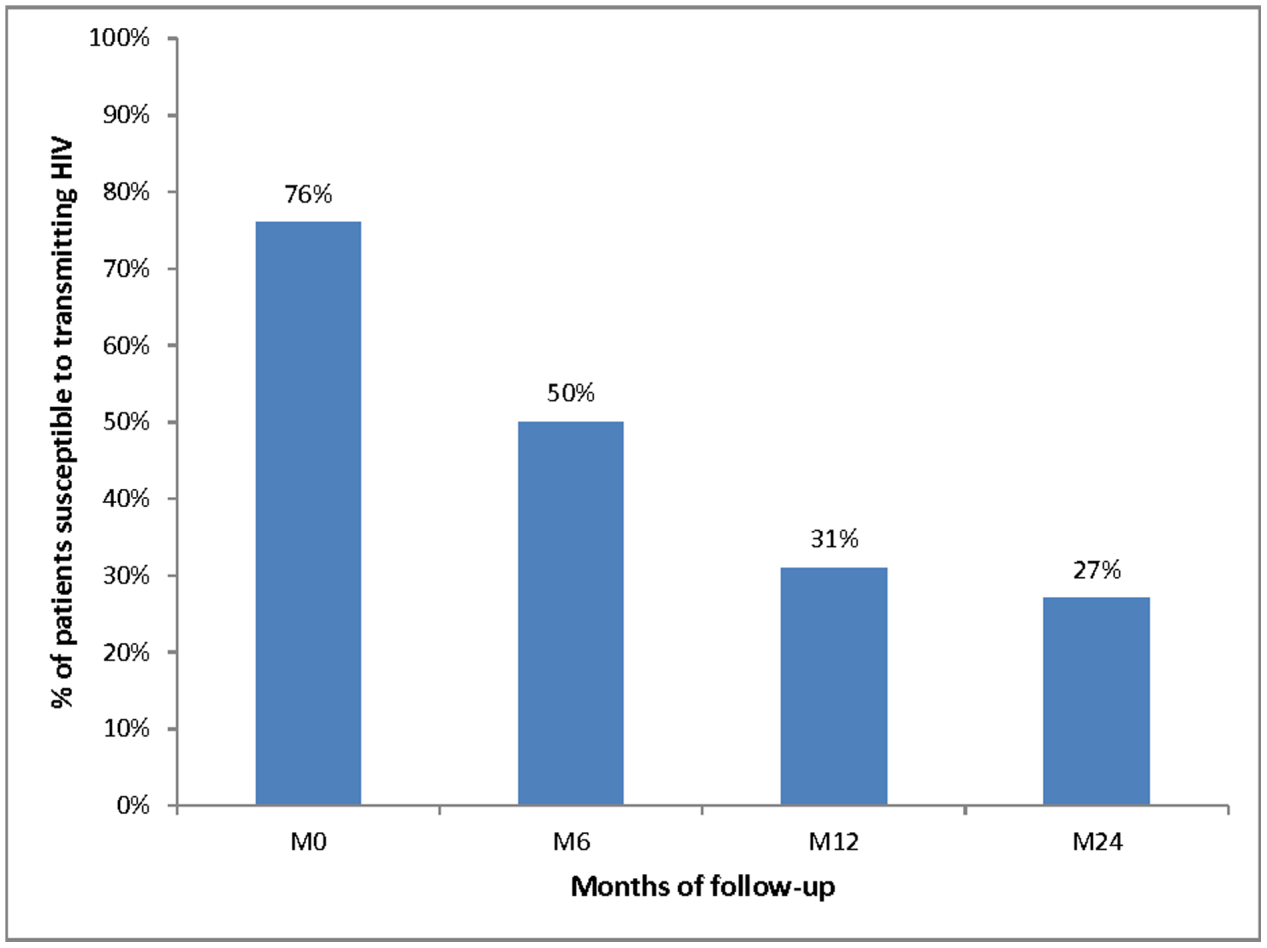
Time trend of the proportion of patients susceptible to transmitting HIV (i.e. reporting both inconsistent condom use and incomplete virological suppression) over the first 24 months of ART (Stratall ANRS 12110/ESTHER trial, 250 patients, 473 visits).

The proportions of patients reporting inconsistent condom use, incomplete virological suppression and susceptibility to transmitting HIV (i.e. patients reporting both incomplete virological suppression and inconsistent condom use) are described in **Table 1** and time trends of these three variables are illustrated in **Figure 1**, **Figure 2** and **Figure 3**, respectively.

**Table 1:** Cross tabulation between incomplete virological suppression (defined as having had at least one detectable viral load (≥40 copies/ml) during the previous 6 months) and inconsistent condom use at each follow up time point (Stratall ANRS 12110/ESTHER trial, 250 patients, 473 visits).

	M0 (n = 114)			M6 (n = 112)			M12 (n = 115)			M24 (n = 132)		
	No (%) of patients not reporting ICU	No (%) of patients reporting ICU	Total	No (%) of patients not reporting ICU	No (%) of patients reporting ICU	Total	No (%) of patients not reporting ICU	No (%) of patients reporting ICU	Total	No (%) of patients not reporting ICU	No (%) of patients reporting ICU	Total
No (%) of patients with completeVL suppression (or undetectableVL at M0)*	0 (0%)	0 (0%)	0 (0%)	0 (0%)	0 (0%)	0 (0%)	14 (12.2%)	32 (27.8%)	46 (40.0%)	24 (18.2%	52 (39.4%)	76 (57.6%)
No (%) of patients with incomplete VL suppression (or detectableVL at M0)**	27 (23.7%)	87 (76.3%)	114 (100%)	56 (50%)	56 (50%)	112 (100%)	33 (28.7%)	36 (31.3%)	69 (60.0%)	21 (15.9%)	35 (26.5%)	56 (42.4%)
**Total**	27 (23.7%)	87 (76.3%)	114 (100%)	56 (50%)	56 (50%)	112 (100%)	47 (40.9%)	68 (59.1%)	115 (100%)	45 (34.1%)	87 (65.9%)	132 (100%)

ICU = Inconsistent condom use; VL = Viral Load.

* As viral load suppression was not defined at M0, figures reported at M0 are related to No. (%) of patients having an undetectable Viral Load (HIV RNA <40copies/ml).

** As viral load suppression was not defined at M0, figures reported at M0 are related to No. (%) of patients having a detectable Viral Load (HIV RNA≥40copies/ml).

The proportion of patients reporting inconsistent condom use significantly decreased from 76% (87/114) at M0 to 50% (56/112) at M6 (p<0.001) and then significantly increased to 59% (68/115) and 66% (87/132) at M12 and M24 respectively (p = 0.023). In addition, the proportion of patients with incomplete virological suppression in the previous 6 months was 100% (112/112) at M6, 60% (69/115) at M12 and 42% (56/132) at M24.

**Table** **1** also shows that the proportion of patients reporting inconsistent condom use among patients experiencing incomplete virological suppression was lower than among patients with complete virological suppression at M12 and M24 (i.e. 52% versus 70% at M12 and 62.5% versus 68% at M24). Finally, the proportion of patients susceptible to transmitting HIV significantly decreased over time from 76% (87/114) at M0, to 50% (56/112) at M6, 31% (36/115) at M12 and 26.5% (35/132) at M24 (p<0.001).

### Predictors of the Susceptibility of Transmitting HIV

As shown in **Table 2**, variables significantly associated in univariate analysis with a higher risk of being susceptible of transmitting HIV included the following: living in a couple (OR [95%CI] = 1.80 [1.12–2.91]), perceiving one’s social level as low (OR [95%CI] = 2.13 [1.28–3.56]), perceiving healthcare staff’s readiness to listen as poor (OR [95%CI] = 2.45 [1.44–4.19]), reporting to have sexual relationships more than once per week (OR [95% CI] = 2.54 [1.46–4.42]), having had more than one sexual partner during the three previous months (OR [95%CI] = 2.66 [1.40–5.05]), experiencing depressive symptoms (OR [95%CI] = 2.26 [1.41–3.61]) and reporting a high number of ART-related symptoms (OR [95% CI] = 1.10 [1.04–1.15]). Conversely, a longer time since ART initiation (OR [95%CI] = 0.55 [0.46–0.65]) for an extra 6 months), reporting moderate to high levels of ART adherence (OR [95%CI] = 0.14 [0.08–0.25])) as well as low adherence (OR [95%CI] = 0.33 [0.14–0.82]) compared with baseline, were all significantly associated with a lower risk of being susceptible to transmitting HIV.

**Table 2:** Factors associated with the susceptibility to transmitting HIV during the first 24 months of antiretroviral therapy in Cameroon: univariate and multivariate analyses using mixed-effects logistic models (Stratall ANRS 12110/ESTHER trial, 250 patients, 473 visits).

Variables	Number of visits (%)or median [IQR]	Number of patients	OR[95% CI]	p-value	AOR[95% CI]	p-value
**Socio-demographic and economic characteristics**						
Age^a^	35 [30]–[43]	−	0.95 [0.74–1.23]	0.70	1.09 [0.79–1.52]	0.59
Female gender	334 (71)	179	1.07 [0.64–1.76]	0.80	1.24 [0.67–2.33]	0.50
Educational level > primary school	252 (53)	129	1.04 [0.65–1.66]	0.87		
Living in a couple	173 (37)	100	1.80 [1.12–2.91]	0.02		
Perceiving one’s social level as low^b^	122 (26)	83	2.13 [1.28–3.56]	0.004		
**Patient-caregiver relationships**						
Having little or no trust in physicians	32 (7)	28	1.53 [0.66–3.51]	0.32		
Having little or no trust in other healthcare staffinvolved in medical follow-up	34 (7)	26	1.57 [0.67–3.68]	0.29		
Perceiving healthcare staff’s readiness to listen as low^c^	123 (26)	99	2.45 [1.44–4.19]	0.001	1.87 [1.01–3.46]	0.04
**Reproductive life and sexual behaviours**						
More than one sexual relationship per week	79(17)	67	2.54 [1.46–4.42]	0.001	2.52 (1.29–4.93]	0.007
More than one sexual partner	65 (14)	53	2.66 [1.40–5.05]	0.003	2.53 [1.21–5.30]	0.01
History of forced sexual relationships	83 (18)	36	1.08 [0.57–2.04]	0.82		
Having at least one biological child	389 (82)	211	1.17 [0.64–2.14]	0.61		
Desire to have a/another child	96 (20)	73	1.51 [0.89–2.57]	0.13	2.07 [1.10–3.87]	0.02
**Psychosocial characteristics**						
Binge drinking	86 (18)	56	1.30 [0.75–2.27]	0.35		
Depressive symptoms^d^	175 (37)	123	2.26 [1.41–3.61]	0.001		
**Clinical and ART-related characteristics**						
Total no. of ART self-reported symptoms +	5 [2]–[9]	−	1.10 [1.04–1.15)	<0.001		
Time since ART initiation (months) ++	12 [6]–[24]	−	0.55 [0.46–0.65]	<0.001	0.66 [0.53–0.83]	<0.001
ART treatment and adherence						
− *Not treated (ref.)*	116 (24)	115	1		1	
−*Treated and non-adherent*	32 (7)	32	0.33 [0.14–0.82]	0.02	0.76 [0.23–2.47]	0.64
−*Treated and adherent*	325 (69)	199	0.14 [0.08–0.25]	<0.001	0.33 [0.15–0.72]	0.005
WHO clinical Stage IV	122 (26)	71	1.04 [0.62–1.75]	0.88		
Intervention group						
*CLIN*	232 (49)	125	1		1	
*LAB*	241 (51)	125	1.19 [0.76–1.88]	0.45	1.15 [0.68–1.95]	0.59

OR = odds ratio; AOR = adjusted odds ratio; IQR = interquartile range; CLIN = clinical monitoring alone; LAB = clinical and laboratory monitoring.

^a^OR per 10-year increase;

b Level 1 or 2 on a ten-point scale;

c Level 1 to 5 on a 6-point visual scale;

d CES-D score>16.

+OR per one-symptom addition,

++OR for an extra 6 months on ART.

Most of these results were confirmed in multivariate analysis (**Table 2**). After adjustment for gender, age and intervention group (clinical versus clinical and laboratory monitoring), we found that perceiving health staff’s readiness to listen as poor (AOR [95% CI] = 1.87 [1.01–3.46]), reporting to have sexual relationships more than once per week (AOR [95% CI] = 2.52 [1.29–4.93]), having had more than one sexual partner during the previous three months (AOR [95% CI] = 2.53 [1.21–5.30]) and desiring to have a/another child (AOR [95% CI] = 2.07 [1.10–3.87]) were all associated with a higher risk of being susceptible to transmitting HIV. In addition, reporting a longer time since ART initiation (AOR [95% CI] = 0.66 [0.53–0.83] for an extra 6 months) and a moderate to high level of adherence (AOR [95% CI] = 0.33 (0.15–0.72]) were significantly associated with a lower risk of being susceptible to transmitting HIV.

## Discussion

In this study, carried out among HIV-infected patients initiating ART in nine rural district hospitals in Cameroon, approximately six out of ten patients were sexually active during follow-up, which is consistent with previous studies showing proportions of sexual activity of about 50% in ART-treated patients in sub-Saharan African countries [15]–[19], [21].

Our findings highlight that the proportion of inconsistent condom use among patients reporting sexual relationships with HIV-negative or unknown HIV status partners dropped sharply in the first six months of treatment but then significantly increased until month 24. Nevertheless, the proportion of patients susceptible to transmitting HIV (i.e. both reporting inconsistent condom use and having had at least one detectable viral load during the previous six months) decreased linearly over the whole follow-up because of a significant decrease in incomplete virological suppression between M12 and M24 which offset the increase in inconsistent condom use observed at the same time points.

To our knowledge, this study is the first to simultaneously investigate risky sexual behaviours on the basis of condom use and incomplete viral suppression in sub-Saharan African settings and accordingly, is the first to attempt to assess HIV transmission risk and its predictors more accurately. From a public health point of view, the decrease in the susceptibility to transmitting HIV highlighted in this study suggests that the risk of behavioural disinhibition following ART initiation should not be a barrier to universal access to ART in the sub-Saharan African setting. Improved access to ART for PLWHA in high HIV prevalence countries could indeed lead to a significant decrease in HIV transmission risk and, in turn, could contribute to control the epidemic.

Although the susceptibility to transmitting HIV decreased during the follow-up, the time trend in inconsistent condom use among PLWHA over the first 24 months of ART showed that the rate of unprotected sex remained at relatively high levels in the study population. The initial drop over the first six months of treatment (from 76% to 50%) was then followed by a constant increase until month 24 of treatment (59% at M12 and 66% at M24), even though the rate of unprotected sex was still lower at M24 than at baseline. The early decrease may be explained both by the impact of prevention counselling at entry into care [14], [35], which may have increased patients’ awareness of their infectiousness [36], [37], and by psychosocial support and adherence counselling which may have synergistic effects on changes in risky behaviours [21]. After the first months of ART, it seems that the early positive impact of counselling was offset by the indirect negative effect of improvement in patients’ health status, which has been shown to be associated with a resumption of sexual activity and a decrease in the perceived risk of HIV transmission [38], [39].

Our results are consistent with those previously reported in studies conducted in sub-Saharan Africa which highlighted a reduction in the rate of inconsistent condom use in the first months after ART initiation [8], [14], [15], [19], [20]. However, the present study is among the first to examine unprotected sex over a longer time period and to highlight an upward time trend [16]. Our results highlight the necessity of continuing preventive counselling over the long term in order to prevent a resurge in inconsistent condom use over follow-up.

This study also contributes to the identification of predictors of the susceptibility to transmitting HIV. First, the study highlights the significant impact of patients’ perception of healthcare staff’s readiness to listen on susceptibility to transmitting HIV, with a near two-fold risk being seen when patients perceived this readiness as poor. Previous studies conducted in Cameroon, including using data from the Stratall trial, have shown that patients’ satisfaction with information provided about treatment and healthcare staff’s readiness to listen was independently associated with a lower risk of non-adherence to ART and inconsistent condom use [33], [40], [41]. In this study, the fact that patients’ perception of healthcare staff’s readiness to listen was also independently associated with the susceptibility to transmitting HIV, even after controlling for ART adherence, suggests that patients who perceived healthcare staff’s readiness to listen as very good would be more receptive and compliant to HIV prevention counselling.

It should be noted that healthcare staff in Cameroon, just as in most sub-Saharan African countries, often face heavy workloads due to shortages in qualified human resources. Heavy workloads however leave no time to listen to patients’ difficulties and provide them with adequate information [26], [42], [43]. Studies conducted on other diseases with long-term treatment, for example tuberculosis [44], [45], highlighted similar difficulties between patients and healthcare workers. They also showed that doctors were more ready to listen to patients’ concerns about strictly medical-related issues, such as the side-effects of medication, than listening to difficulties which were more personal in nature (e.g. related to treatment adherence, lack of family support or emotional distress). This latter result highlights the need both to reduce professionals’ HIV care workload through task-shifting and to strengthen caregivers’ communication skills, in order to ensure adequate counselling.

Second, adherence to ART was unsurprisingly found to be independently associated with a lower risk of being susceptible to transmitting HIV. Indeed, it is well known that patients with low adherence are at a higher risk of virological failure [46]–[48]. Furthermore a previous study conducted using Stratall data has demonstrated a relationship between inconsistent condom use and low adherence behaviours which both may be considered as “risky” behaviours [33]. Another study conducted in United States has also shown that individuals who engage in risky behaviours also report poorer ART adherence [32]. This result suggests that integrated behavioural interventions to improve ART adherence could also be beneficial in terms of the susceptibility to transmitting HIV both through a direct impact on viremia reduction and through a decrease in unprotected sex and risk compensation beliefs.

Third, the study’s findings show that patients who desired to have children were also more susceptible to transmitting HIV. The desire to have children is a well-known factor of inconsistent condom use [21], [33]. However the fact that it is also a predictor of the susceptibility to transmitting HIV highlights even more the urgent need to design and propose adequate interventions for HIV-infected individuals in serodiscordant couples who desire to have children. This would include interventions to encourage HIV disclosure to the main sexual partner, to support patients in this process and to provide adequate counselling to couples about safer ways to procreate, such as 1) controlling viremia before trying to conceive, 2) limiting unprotected relationships during the period of fertilization, if viremia is controlled, and 3) using antiretroviral therapy chemoprophylaxis for the uninfected at-risk partner [49], [50].

Finally, it is worth noting that predictors both of the susceptibility to transmitting HIV and inconsistent condom use also include the frequency of sexual intercourse and the number of sexual partners.

Despite the importance of the study findings, some limitations should be acknowledged. First, perception of the patient-caregiver relationship, adherence and sexual behaviour assessment were based on self-reports, which are known to be affected by social desirability bias [51]. Adherence counselling and preventive messages about condom use may have resulted in under-declaration of non-adherence and of risky sexual behaviours. Likewise dissatisfaction with the patient-caregiver relationship may have been underreported. However, as interviews in both groups were conducted by the same interviewers, there is no risk that information was biased differently between the two randomized groups. In addition, in terms of adherence measurement, we minimized the risk of bias by using a specific algorithm, computed using several questions, which showed good correlation with viremia [30], [31]. We also minimized possible face-to-face interview-induced social desirability bias by training community healthcare workers to use non-judgmental approaches during interviews so that patients felt comfortable when answering questions. In addition, interview confidentiality, including medical staff confidentiality, was guaranteed. Another limitation is related to the fact that the study did not collect information about the different types of casual partners, for example sex-trade partners. Although condom use with sex-trade partners may be reported very differently than for other types of partnerships, we were unable to take into account the specific effect of having sex-trade partners on the susceptibility to transmitting HIV. Finally, the study’s results are limited by the 24-month duration of the follow-up. Future studies are needed to examine how sexual risk behaviours will evolve over longer periods of follow-up and how long the decrease in the susceptibility to transmitting HIV observed over the first 24 months of treatment will be maintained, given that the prevalence of treatment failure is expected to increase over time.

In conclusion, although the concept of “susceptibility to transmitting HIV” should be further explored in others settings and populations, the results from this study suggest that fear of behavioural disinhibition should not be a barrier to universal access to ART in sub-Saharan African countries. Expanding access to ART to all HIV-infected individuals in this region while promoting HIV risk prevention interventions could lead to a dramatic decrease in HIV transmission. However, this will only be successful through significant capacity building of healthcare staff’s counselling skills and adequate interventions matching patients’ expectations and needs like the desire to have children in serodiscordant couples.
