# Correction: Susceptibility to Transmitting HIV in Patients Initiating Antiretroviral Therapy in Rural District Hospitals in Cameroon (Stratall ANRS 12110/ESTHER Trial)

**DOI:** 10.1371/annotation/8963fb4d-336a-4607-8fd9-c3404414ff9c

**Published:** 2013-07-08

**Authors:** Gilbert Ndziessi, Julien Cohen, Charles Kouanfack, Fabienne Marcellin, Maria Patrizia Carrieri, Gabrièle Laborde-Balen, Camélia Protopopescu, Avelin Fobang Aghokeng, Jean-Paul Moatti, Bruno Spire, Eric Delaporte, Christian Laurent, Sylvie Boyer, for the Stratall ANRS 12110 / ESTHER Study Group, M. Biwolé-Sida, C. Kouanfack, S. Koulla-Shiro, A. Bourgeois, E. Delaporte, C. Laurent, M. Peeters, G. Laborde-Balen, M. Dontsop, S. Kazé, J-M. Mben, A. Aghokeng, M.G. Edoul, E. Mpoudi-Ngolé, M. Tongo, S. Boyer, M.P. Carrieri, F. Marcellin, J-P. Moatti, B. Spire, C. Abé, S-C. Abega, C-R. Bonono, H. Mimcheu, S. Ngo Yebga, C. Paul Bile, S. Abada, T. Abanda, J. Baga, P. Bilobi Fouda, P. Etong Mve, G. Fetse Tama, H. Kemo, A. Ongodo, V. Tadewa, HD. Voundi, A. Ambani, M. Atangana, J-C. Biaback, M. Kennedy, H. Kibedou, F. Kounga, M. Maguip Abanda, E. Mamang, A. Mikone, S. Tang, E. Tchuangue, S. Tchuenko, D. Yakan, J. Assandje, S. Ebana, D. Ebo’o, D. Etoundi, G. Ngama, P. Mbarga Ango, J. Mbezele, G. Mbong, C. Moung, N. Ekotto, G. Nguemba Balla, G. Ottou, M. Tigougmo, R. Beyala, B. Ebene, C. Effemba, F. Eyebe, M-M. Hadjaratou, T. Mbarga, M. Metou, M. Ndam, B. Ngoa, EB. Ngock, N. Obam, A. M. Abomo, G. Angoula, E. Ekassi, Essama, J.J. Lentchou, I. Mvilongo, J. Ngapou, F. Ntokombo, V. Ondoua, R. Palawo, S. Sebe, E. Sinou, D. Wankam, I. Zobo, B. Akono, A. L. Ambani, L. Bilock, R. Bilo’o, J. Boombhi, F.X. Fouda, M. Guitonga, R. Mad’aa, D.R. Metou’ou, S. Mgbih, A. Noah, M. Tadena, Ntcham, G. Ambassa Elime, A.A. Bonongnaba, E. Foaleng, R.M. Heles, R. Messina, O. Nana Ndankou, S.A. Ngono, D. Ngono Menounga, S.S. Sil, L. Tchouamou, B. Zambou, R. Abomo, J. Ambomo, C. Beyomo, P. Eloundou, C. Ewole, J. Fokom, M. Mvoto, M. Ngadena, R. Nyolo, C. Onana, A. Oyie, P. Antyimi, S. Bella Mbatonga, M. Bikomo, Y. Molo Bodo, S. Ndi Ntang, P. Ndoudoumou, L. Ndzomo, S.O. Ngolo, M. Nkengue, Nkoa, Y. Tchinda

**Affiliations:** Central hospital, Yaoundé, Cameroon; Central hospital, Yaoundé, Cameroon; Central hospital, Yaoundé, Cameroon; IRD, University Montpellier 1, UMI 233, Montpellier, France; IRD, University Montpellier 1, UMI 233, Montpellier, France; IRD, University Montpellier 1, UMI 233, Montpellier, France; IRD, University Montpellier 1, UMI 233, Montpellier, France; French Ministry of Foreign Affairs, Yaoundé, Cameroon; IRD, Yaoundé, Cameroon; IRD, Yaoundé, Cameroon; IRD, Yaoundé, Cameroon; Virology Laboratory, IMPM/CREMER/IRD-UMI 233, Yaoundé, Cameroon; Virology Laboratory, IMPM/CREMER/IRD-UMI 233, Yaoundé, Cameroon; Virology Laboratory, IMPM/CREMER/IRD-UMI 233, Yaoundé, Cameroon; Virology Laboratory, IMPM/CREMER/IRD-UMI 233, Yaoundé, Cameroon; INSERM, IRD, University Marseille, UMR 912, Marseille, France; INSERM, IRD, University Marseille, UMR 912, Marseille, France; INSERM, IRD, University Marseille, UMR 912, Marseille, France; INSERM, IRD, University Marseille, UMR 912, Marseille, France; INSERM, IRD, University Marseille, UMR 912, Marseille, France; IRSA, Catholic University of Central Africa, Yaoundé, Cameroon; IRSA, Catholic University of Central Africa, Yaoundé, Cameroon; IRSA, Catholic University of Central Africa, Yaoundé, Cameroon; IRSA, Catholic University of Central Africa, Yaoundé, Cameroon; IRSA, Catholic University of Central Africa, Yaoundé, Cameroon; IRSA, Catholic University of Central Africa, Yaoundé, Cameroon; District Hospital, Ayos, Cameroon; District Hospital, Ayos, Cameroon; District Hospital, Ayos, Cameroon; District Hospital, Ayos, Cameroon; District Hospital, Ayos, Cameroon; District Hospital, Ayos, Cameroon; District Hospital, Ayos, Cameroon; District Hospital, Ayos, Cameroon; District Hospital, Ayos, Cameroon; District Hospital, Ayos, Cameroon; District Hospital, Bafia, Cameroon; District Hospital, Bafia, Cameroon; District Hospital, Bafia, Cameroon; District Hospital, Bafia, Cameroon; District Hospital, Bafia, Cameroon; District Hospital, Bafia, Cameroon; District Hospital, Bafia, Cameroon; District Hospital, Bafia, Cameroon; District Hospital, Bafia, Cameroon; District Hospital, Bafia, Cameroon; District Hospital, Bafia, Cameroon; District Hospital, Bafia, Cameroon; District Hospital, Bafia, Cameroon; District Hospital, Mbalmayo, Cameroon; District Hospital, Mbalmayo, Cameroon; District Hospital, Mbalmayo, Cameroon; District Hospital, Mbalmayo, Cameroon; District Hospital, Mbalmayo, Cameroon; District Hospital, Mbalmayo, Cameroon; District Hospital, Mbalmayo, Cameroon; District Hospital, Mbalmayo, Cameroon; District Hospital, Mbalmayo, Cameroon; District Hospital, Mbalmayo, Cameroon; District Hospital, Mbalmayo, Cameroon; District Hospital, Mbalmayo, Cameroon; District Hospital, Mbalmayo, Cameroon; District hospital, Mfou, Cameroon; District hospital, Mfou, Cameroon; District hospital, Mfou, Cameroon; District hospital, Mfou, Cameroon; District hospital, Mfou, Cameroon; District hospital, Mfou, Cameroon; District hospital, Mfou, Cameroon; District hospital, Mfou, Cameroon; District hospital, Mfou, Cameroon; District hospital, Mfou, Cameroon; District hospital, Mfou, Cameroon; District hospital, Monatélé, Cameroon; District hospital, Monatélé, Cameroon; District hospital, Monatélé, Cameroon; District hospital, Monatélé, Cameroon; District hospital, Monatélé, Cameroon; District hospital, Monatélé, Cameroon; District hospital, Monatélé, Cameroon; District hospital, Monatélé, Cameroon; District hospital, Monatélé, Cameroon; District hospital, Monatélé, Cameroon; District hospital, Monatélé, Cameroon; District hospital, Monatélé, Cameroon; District hospital, Monatélé, Cameroon; District hospital, Monatélé, Cameroon; District hospital, Nanga Eboko, Cameroon; District hospital, Nanga Eboko, Cameroon; District hospital, Nanga Eboko, Cameroon; District hospital, Nanga Eboko, Cameroon; District hospital, Nanga Eboko, Cameroon; District hospital, Nanga Eboko, Cameroon; District hospital, Nanga Eboko, Cameroon; District hospital, Nanga Eboko, Cameroon; District hospital, Nanga Eboko, Cameroon; District hospital, Nanga Eboko, Cameroon; District hospital, Nanga Eboko, Cameroon; District hospital, Nanga Eboko, Cameroon; District hospital, Nanga Eboko, Cameroon; District hospital, Ndikinimeki, Cameroon; District hospital, Ndikinimeki, Cameroon; District hospital, Ndikinimeki, Cameroon; District hospital, Ndikinimeki, Cameroon; District hospital, Ndikinimeki, Cameroon; District hospital, Ndikinimeki, Cameroon; District hospital, Ndikinimeki, Cameroon; District hospital, Ndikinimeki, Cameroon; District hospital, Ndikinimeki, Cameroon; District hospital, Ndikinimeki, Cameroon; District hospital, Ndikinimeki, Cameroon; District hospital, Obala, Cameroon; District hospital, Obala, Cameroon; District hospital, Obala, Cameroon; District hospital, Obala, Cameroon; District hospital, Obala, Cameroon; District hospital, Obala, Cameroon; District hospital, Obala, Cameroon; District hospital, Obala, Cameroon; District hospital, Obala, Cameroon; District hospital, Obala, Cameroon; District hospital, Obala, Cameroon; District hospital, Sa’a, Cameroon; District hospital, Sa’a, Cameroon; District hospital, Sa’a, Cameroon; District hospital, Sa’a, Cameroon; District hospital, Sa’a, Cameroon; District hospital, Sa’a, Cameroon; District hospital, Sa’a, Cameroon; District hospital, Sa’a, Cameroon; District hospital, Sa’a, Cameroon; District hospital, Sa’a, Cameroon; District hospital, Sa’a, Cameroon

The name of the fifth author was spelled incorrectly. The correct name is: Maria Patrizia Carrieri. The correct citation is: Ndziessi G, Cohen J, Kouanfack C, Marcellin F, Carrieri MP, et al. (2013) Susceptibility to Transmitting HIV in Patients Initiating Antiretroviral Therapy in Rural District Hospitals in Cameroon (Stratall ANRS 12110/ESTHER Trial). PLoS ONE 8(4): e62611. doi:10.1371/journal.pone.0062611. 

