# Correction: Susceptibility to Transmitting HIV in Patients Initiating Antiretroviral Therapy in Rural District Hospitals in Cameroon (Stratall ANRS 12110/ESTHER Trial)

**DOI:** 10.1371/journal.pone.0323690

**Published:** 2025-05-08

**Authors:** Gilbert Ndziessi, Julien Cohen, Charles Kouanfack, Fabienne Marcellin, Maria Patrizia Carierri, Gabrièle Laborde-Balen, Camélia Protopopescu, Avelin Fobang Aghokeng, Jean-Paul Moatti, Bruno Spire, Eric Delaporte, Christian Laurent, Sylvie Boyer, for the Stratall ANRS 12110/ ESTHER Study Group

The name of a member of the Stratall ANRS 12–110/ESTHER Study Group was incorrectly listed as J. Fokom. The correct name is Joël Fokom Domgue.

The corrected Acknowledgments should read: The authors thank the Ministry of Public Health in Cameroon, as well as the patients and staff from all hospitals participating in the Stratall trial. We also thank Jude Sweeney (Rome, Italy) for the English revision and editing of the manuscript. Data presented previously in oral communication at Sixth International AIDS Society Conference on HIV Pathogenesis, Treatment and Prevention, Rome, July 17–20, 2011 [abstract MOAC0203].

## Stratall ANRS 12110/ESTHER study group

M. Biwolé-Sida C., Kouanfack, S. Koulla-Shiro (Central hospital, Yaoundé, Cameroon); A. Bourgeois, E. Delaporte, C. Laurent, M. Peeters (IRD, University Montpellier 1, UMI 233, Montpellier, France); G. Laborde-Balen (French Ministry of Foreign Affairs, Yaoundé, Cameroon); M. Dontsop, S. Kazé, J-M. Mben (IRD, Yaoundé, Cameroon); A. Aghokeng, M.G. Edoul, E. Mpoudi-Ngolé, M. Tongo (Virology Laboratory, IMPM/CREMER/IRD-UMI 233, Yaoundé, Cameroon); S. Boyer, M.P. Carrieri, F. Marcellin, J-P. Moatti, B. Spire (INSERM, IRD, University Marseille, UMR 912, Marseille, France); C. Abé, S-C. Abega, C-R. Bonono, H. Mimcheu, S. Ngo Yebga, C. Paul Bile (IRSA, Catholic University of Central Africa, Yaoundé, Cameroon); S. Abada, T. Abanda, J. Baga, P. Bilobi Fouda, P. Etong Mve, G. Fetse Tama, H. Kemo, A. Ongodo, V. Tadewa, HD. Voundi (District Hospital, Ayos, Cameroon); A. Ambani, M. Atangana, J-C. Biaback, M. Kennedy, H. Kibedou, F. Kounga, M. Maguip Abanda, E. Mamang, A. Mikone, S. Tang, E. Tchuangue, S. Tchuenko, D. Yakan (District Hospital, Bafia, Cameroon); J. Assandje, S. Ebana, D. Ebo’o, D. Etoundi, G. Ngama, P. Mbarga Ango, J. Mbezele, G. Mbong, C. Moung, N. Ekotto, G. Nguemba Balla, G. Ottou, M. Tigougmo (District Hospital, Mbalmayo, Cameroon); R. Beyala, B. Ebene, C. Effemba, F. Eyebe, M-M. Hadjaratou, T. Mbarga, M. Metou, M. Ndam, B. Ngoa, EB. Ngock, N. Obam (District hospital, Mfou, Cameroon); A. M. Abomo, G. Angoula, E. Ekassi, Essama, J.J. Lentchou, I. Mvilongo, J. Ngapou, F. Ntokombo, V. Ondoua, R. Palawo, S. Sebe, E. Sinou, D. Wankam, I. Zobo (District hospital, Monatélé, Cameroon); B. Akono, A. L. Ambani, L. Bilock, R. Bilo’o, J. Boombhi, F.X. Fouda, M. Guitonga, R. Mad’aa, D.R. Metou’ou, S. Mgbih, A. Noah, M. Tadena, Ntcham (District hospital, Nanga Eboko, Cameroon); G. Ambassa Elime, A.A. Bonongnaba, E. Foaleng, R.M. Heles, R. Messina, O. Nana Ndankou, S.A. Ngono, D. Ngono Menounga, S.S. Sil, L. Tchouamou, B. Zambou (District hospital, Ndikinimeki, Cameroon); R. Abomo, J. Ambomo, C. Beyomo, P. Eloundou, C. Ewole, Joël Fokom Domgue, M. Mvoto, M. Ngadena, R. Nyolo, C. Onana, A. Oyie (District hospital, Obala, Cameroon); P. Antyimi, S. Bella Mbatonga, M. Bikomo, Y. Molo Bodo, S. Ndi Ntang, P. Ndoudoumou, L. Ndzomo, S.O. Ngolo, M. Nkengue, Nkoa, Y. Tchinda (District hospital, Sa’a, Cameroon).
